# Supplementary material for: OpenMM 7: Rapid development of high performance algorithms for molecular dynamics
Source: PLoS Comput Biol. 2017 Jul 26;13(7):e1005659. doi: 10.1371/journal.pcbi.1005659 (PMC5549999; doi:10.1371/journal.pcbi.1005659)
Supplement: S1 Source Code — (ZIP) [file pcbi.1005659.s002.zip › OpenMM-7.0.1-Source/docs/api-c++/generated/OpenMM.AmoebaStretchBendForce.html]

AmoebaStretchBendForce — OpenMM @OPENMM\_MAJOR\_VERSION@.@OPENMM\_MINOR\_VERSION@ documentation


# AmoebaStretchBendForce¶

*class* `OpenMM::``AmoebaStretchBendForce`¶
:   This class implements the Amoeba stretch-bend interaction.

    To use it, create a StretchBendForce object then call `addStretchBend()` once for each stretch-bend. After a stretch-bend has been added, you can modify its force field parameters by calling `setStretchBendParameters()`. This will have no effect on Contexts that already exist unless you call `updateParametersInContext()`.

    Methods

    |  |  |
    | --- | --- |
    | `AmoebaStretchBendForce` | Create an `AmoebaStretchBendForce`. |
    | `getNumStretchBends` | Get the number of stretch-bend terms in the potential function |
    | `addStretchBend` | Add a stretch-bend term to the force field. |
    | `getStretchBendParameters` | Get the force field parameters for a stretch-bend term. |
    | `setStretchBendParameters` | Set the force field parameters for a stretch-bend term. |
    | `updateParametersInContext` | Update the per-stretch-bend term parameters in a `Context` to match those stored in this `Force` object. |
    | `usesPeriodicBoundaryConditions` | Returns whether or not this force makes use of periodic boundary conditions. |

    `AmoebaStretchBendForce`()¶
    :   Create an `AmoebaStretchBendForce`.

    int `getNumStretchBends`() *const*¶
    :   Get the number of stretch-bend terms in the potential function

    int `addStretchBend`(int *particle1*, int *particle2*, int *particle3*, double *lengthAB*, double *lengthCB*, double *angle*, double *k1*, double *k2*)¶
    :   Add a stretch-bend term to the force field.

        |  |  |
        | --- | --- |
        | Parameters: |  |

        - **particle1** – the index of the first particle connected by the stretch-bend
        - **particle2** – the index of the second particle connected by the stretch-bend
        - **particle3** – the index of the third particle connected by the stretch-bend
        - **lengthAB** – the equilibrium length of the stretch-bend in bond ab [particle1, particle2], measured in nm
        - **lengthCB** – the equilibrium length of the stretch-bend in bond cb [particle3, particle2], measured in nm
        - **angle** – the equilibrium angle in radians
        - **k1** – the force constant of the product of bond ab and angle a-b-c
        - **k2** – the force constant of the product of bond bc and angle a-b-c (optional, default is the same as k1)

        |  |  |
        | --- | --- |
        | Returns: | the index of the stretch-bend that was added |

    void `getStretchBendParameters`(int *index*, int &*particle1*, int &*particle2*, int &*particle3*, double &*lengthAB*, double &*lengthCB*, double &*angle*, double &*k1*, double &*k2*) *const*¶
    :   Get the force field parameters for a stretch-bend term.

        |  |  |
        | --- | --- |
        | Parameters: |  |

        - **index** – the index of the stretch-bend for which to get parameters
        - **particle1** – [out] the index of the first particle connected by the stretch-bend
        - **particle2** – [out] the index of the second particle connected by the stretch-bend
        - **particle3** – [out] the index of the third particle connected by the stretch-bend
        - **lengthAB** – [out] the equilibrium length of the stretch-bend in bond ab [particle1, particle2], measured in nm
        - **lengthCB** – [out] the equilibrium length of the stretch-bend in bond cb [particle3, particle2], measured in nm
        - **angle** – [out] the equilibrium angle in radians
        - **k1** – [out] the force constant of the product of bond ab and angle a-b-c
        - **k2** – [out] the force constant of the product of bond bc and angle a-b-c

    void `setStretchBendParameters`(int *index*, int *particle1*, int *particle2*, int *particle3*, double *lengthAB*, double *lengthCB*, double *angle*, double *k1*, double *k2*)¶
    :   Set the force field parameters for a stretch-bend term.

        |  |  |
        | --- | --- |
        | Parameters: |  |

        - **index** – the index of the stretch-bend for which to set parameters
        - **particle1** – the index of the first particle connected by the stretch-bend
        - **particle2** – the index of the second particle connected by the stretch-bend
        - **particle3** – the index of the third particle connected by the stretch-bend
        - **lengthAB** – the equilibrium length of the stretch-bend in bond ab [particle1, particle2], measured in nm
        - **lengthCB** – the equilibrium length of the stretch-bend in bond cb [particle3, particle2], measured in nm
        - **angle** – the equilibrium angle in radians
        - **k1** – the force constant of the product of bond ab and angle a-b-c
        - **k2** – the force constant of the product of bond bc and angle a-b-c (optional, default is the same as k1)

    void `updateParametersInContext`(Context &*context*)¶
    :   Update the per-stretch-bend term parameters in a `Context` to match those stored in this `Force` object. This method provides an efficient method to update certain parameters in an existing `Context` without needing to reinitialize it. Simply call `setStretchBendParameters()` to modify this object’s parameters, then call `updateParametersInContext()` to copy them over to the `Context`.

        The only information this method updates is the values of per-stretch-bend term parameters. The set of particles involved in a term cannot be changed, nor can new terms be added.

    bool `usesPeriodicBoundaryConditions`() *const*¶
    :   Returns whether or not this force makes use of periodic boundary conditions.

        |  |  |
        | --- | --- |
        | Returns: | true if nonbondedMethod uses PBC and false otherwise |

High performance molecular simulation on GPUs


### Navigation

- Core Objects
- Forces
- Integrators
- Extras

©2015, Stanford University and the Authors.
|
Powered by Sphinx 1.3.1
& Alabaster 0.7.7
|
Page source
